# Supplementary material for: The Gut Bacteria Dysbiosis Contributes to Chronic Graft-Versus-Host Disease Associated With a Treg/Th1 Ratio Imbalance
Source: Front Microbiol. 2022 Sep 8;13:813576. doi: 10.3389/fmicb.2022.813576 (PMC9493085; doi:10.3389/fmicb.2022.813576)
Supplement: Supplementary file 1 [file Data_Sheet_1.zip › P101SC18090073-01-B1-3-4_result/05.WebShow/taxa_charts_group_html/area_charts.html]

Taxa Summaries


|  |  |
| --- | --- |
|  | |
| Taxonomy Summary. Current Level: | |
| View Figure (.pdf)  View Legend (.pdf) |  |
|  |  |


View Table (.txt)

|  |  |  |  |  |  |
| --- | --- | --- | --- | --- | --- |
|  | | Total | | MD | MC |
| Legend | Taxonomy | count | % | % | % |
|  | k\_\_Archaea;p\_\_Euryarchaeota | 0 | 0.0% | 0.0% | 0.0% |
|  | k\_\_Bacteria;Other | 0 | 0.1% | 0.1% | 0.1% |
|  | k\_\_Bacteria;p\_\_Acidobacteria | 0 | 0.0% | 0.0% | 0.0% |
|  | k\_\_Bacteria;p\_\_Actinobacteria | 0 | 0.0% | 0.0% | 0.1% |
|  | k\_\_Bacteria;p\_\_Armatimonadetes | 0 | 0.0% | 0.0% | 0.0% |
|  | k\_\_Bacteria;p\_\_Bacteroidetes | 1 | 52.8% | 51.2% | 54.5% |
|  | k\_\_Bacteria;p\_\_Deinococcus-Thermus | 0 | 0.0% | 0.0% | 0.0% |
|  | k\_\_Bacteria;p\_\_Firmicutes | 1 | 38.1% | 39.6% | 36.6% |
|  | k\_\_Bacteria;p\_\_Fusobacteria | 0 | 0.0% | 0.0% | 0.0% |
|  | k\_\_Bacteria;p\_\_Melainabacteria | 0 | 0.0% | 0.0% | 0.1% |
|  | k\_\_Bacteria;p\_\_Oxyphotobacteria | 0 | 0.0% | 0.1% | 0.0% |
|  | k\_\_Bacteria;p\_\_Proteobacteria | 0 | 6.9% | 8.4% | 5.5% |
|  | k\_\_Bacteria;p\_\_Tenericutes | 0 | 0.5% | 0.1% | 0.8% |
|  | k\_\_Bacteria;p\_\_Verrucomicrobia | 0 | 1.4% | 0.4% | 2.3% |
|  | k\_\_Bacteria;p\_\_unidentified\_Bacteria | 0 | 0.1% | 0.0% | 0.1% |

|  |  |
| --- | --- |
|  | |
| Taxonomy Summary. Current Level: | |
| View Figure (.pdf)  View Legend (.pdf) |  |
|  |  |


View Table (.txt)

|  |  |  |  |  |  |
| --- | --- | --- | --- | --- | --- |
|  | | Total | | MD | MC |
| Legend | Taxonomy | count | % | % | % |
|  | k\_\_Archaea;p\_\_Euryarchaeota;c\_\_Methanobacteria | 0 | 0.0% | 0.0% | 0.0% |
|  | k\_\_Bacteria;Other;Other | 0 | 0.1% | 0.1% | 0.1% |
|  | k\_\_Bacteria;p\_\_Acidobacteria;c\_\_Holophagae | 0 | 0.0% | 0.0% | 0.0% |
|  | k\_\_Bacteria;p\_\_Acidobacteria;c\_\_unidentified\_Acidobacteria | 0 | 0.0% | 0.0% | 0.0% |
|  | k\_\_Bacteria;p\_\_Actinobacteria;c\_\_Acidimicrobiia | 0 | 0.0% | 0.0% | 0.0% |
|  | k\_\_Bacteria;p\_\_Actinobacteria;c\_\_Coriobacteriia | 0 | 0.0% | 0.0% | 0.0% |
|  | k\_\_Bacteria;p\_\_Actinobacteria;c\_\_Rubrobacteria | 0 | 0.0% | 0.0% | 0.0% |
|  | k\_\_Bacteria;p\_\_Actinobacteria;c\_\_Thermoleophilia | 0 | 0.0% | 0.0% | 0.0% |
|  | k\_\_Bacteria;p\_\_Actinobacteria;c\_\_unidentified\_Actinobacteria | 0 | 0.0% | 0.0% | 0.0% |
|  | k\_\_Bacteria;p\_\_Armatimonadetes;c\_\_Fimbriimonadia | 0 | 0.0% | 0.0% | 0.0% |
|  | k\_\_Bacteria;p\_\_Bacteroidetes;c\_\_Bacteroidia | 1 | 52.8% | 51.2% | 54.5% |
|  | k\_\_Bacteria;p\_\_Deinococcus-Thermus;c\_\_Deinococci | 0 | 0.0% | 0.0% | 0.0% |
|  | k\_\_Bacteria;p\_\_Firmicutes;Other | 0 | 0.0% | 0.0% | 0.0% |
|  | k\_\_Bacteria;p\_\_Firmicutes;c\_\_Bacilli | 0 | 1.0% | 1.1% | 0.9% |
|  | k\_\_Bacteria;p\_\_Firmicutes;c\_\_Clostridia | 1 | 36.6% | 38.2% | 35.0% |
|  | k\_\_Bacteria;p\_\_Firmicutes;c\_\_Erysipelotrichia | 0 | 0.4% | 0.3% | 0.6% |
|  | k\_\_Bacteria;p\_\_Firmicutes;c\_\_Negativicutes | 0 | 0.0% | 0.0% | 0.1% |
|  | k\_\_Bacteria;p\_\_Fusobacteria;c\_\_Fusobacteriia | 0 | 0.0% | 0.0% | 0.0% |
|  | k\_\_Bacteria;p\_\_Melainabacteria;Other | 0 | 0.0% | 0.0% | 0.1% |
|  | k\_\_Bacteria;p\_\_Oxyphotobacteria;c\_\_unidentified\_Oxyphotobacteria | 0 | 0.0% | 0.1% | 0.0% |
|  | k\_\_Bacteria;p\_\_Proteobacteria;Other | 0 | 0.0% | 0.0% | 0.0% |
|  | k\_\_Bacteria;p\_\_Proteobacteria;c\_\_Alphaproteobacteria | 0 | 0.0% | 0.0% | 0.0% |
|  | k\_\_Bacteria;p\_\_Proteobacteria;c\_\_Deltaproteobacteria | 0 | 1.1% | 1.1% | 1.1% |
|  | k\_\_Bacteria;p\_\_Proteobacteria;c\_\_Gammaproteobacteria | 0 | 5.8% | 7.2% | 4.3% |
|  | k\_\_Bacteria;p\_\_Tenericutes;c\_\_Mollicutes | 0 | 0.5% | 0.1% | 0.8% |
|  | k\_\_Bacteria;p\_\_Verrucomicrobia;c\_\_Verrucomicrobiae | 0 | 1.4% | 0.4% | 2.3% |
|  | k\_\_Bacteria;p\_\_unidentified\_Bacteria;c\_\_unidentified\_Bacteria | 0 | 0.1% | 0.0% | 0.1% |

|  |  |
| --- | --- |
|  | |
| Taxonomy Summary. Current Level: | |
| View Figure (.pdf)  View Legend (.pdf) |  |
|  |  |


View Table (.txt)

|  |  |  |  |  |  |
| --- | --- | --- | --- | --- | --- |
|  | | Total | | MD | MC |
| Legend | Taxonomy | count | % | % | % |
|  | k\_\_Archaea;p\_\_Euryarchaeota;c\_\_Methanobacteria;o\_\_Methanobacteriales | 0 | 0.0% | 0.0% | 0.0% |
|  | k\_\_Bacteria;Other;Other;Other | 0 | 0.1% | 0.1% | 0.1% |
|  | k\_\_Bacteria;p\_\_Acidobacteria;c\_\_Holophagae;o\_\_ | 0 | 0.0% | 0.0% | 0.0% |
|  | k\_\_Bacteria;p\_\_Acidobacteria;c\_\_unidentified\_Acidobacteria;o\_\_unidentified\_Acidobacteria | 0 | 0.0% | 0.0% | 0.0% |
|  | k\_\_Bacteria;p\_\_Actinobacteria;c\_\_Acidimicrobiia;Other | 0 | 0.0% | 0.0% | 0.0% |
|  | k\_\_Bacteria;p\_\_Actinobacteria;c\_\_Acidimicrobiia;o\_\_ | 0 | 0.0% | 0.0% | 0.0% |
|  | k\_\_Bacteria;p\_\_Actinobacteria;c\_\_Coriobacteriia;o\_\_Coriobacteriales | 0 | 0.0% | 0.0% | 0.0% |
|  | k\_\_Bacteria;p\_\_Actinobacteria;c\_\_Rubrobacteria;o\_\_Rubrobacterales | 0 | 0.0% | 0.0% | 0.0% |
|  | k\_\_Bacteria;p\_\_Actinobacteria;c\_\_Thermoleophilia;o\_\_Gaiellales | 0 | 0.0% | 0.0% | 0.0% |
|  | k\_\_Bacteria;p\_\_Actinobacteria;c\_\_Thermoleophilia;o\_\_Solirubrobacterales | 0 | 0.0% | 0.0% | 0.0% |
|  | k\_\_Bacteria;p\_\_Actinobacteria;c\_\_unidentified\_Actinobacteria;o\_\_Actinomycetales | 0 | 0.0% | 0.0% | 0.0% |
|  | k\_\_Bacteria;p\_\_Actinobacteria;c\_\_unidentified\_Actinobacteria;o\_\_Bifidobacteriales | 0 | 0.0% | 0.0% | 0.0% |
|  | k\_\_Bacteria;p\_\_Actinobacteria;c\_\_unidentified\_Actinobacteria;o\_\_Corynebacteriales | 0 | 0.0% | 0.0% | 0.0% |
|  | k\_\_Bacteria;p\_\_Actinobacteria;c\_\_unidentified\_Actinobacteria;o\_\_Micrococcales | 0 | 0.0% | 0.0% | 0.0% |
|  | k\_\_Bacteria;p\_\_Actinobacteria;c\_\_unidentified\_Actinobacteria;o\_\_Propionibacteriales | 0 | 0.0% | 0.0% | 0.0% |
|  | k\_\_Bacteria;p\_\_Armatimonadetes;c\_\_Fimbriimonadia;o\_\_Fimbriimonadales | 0 | 0.0% | 0.0% | 0.0% |
|  | k\_\_Bacteria;p\_\_Bacteroidetes;c\_\_Bacteroidia;o\_\_Bacteroidales | 1 | 52.8% | 51.2% | 54.5% |
|  | k\_\_Bacteria;p\_\_Bacteroidetes;c\_\_Bacteroidia;o\_\_Chitinophagales | 0 | 0.0% | 0.0% | 0.0% |
|  | k\_\_Bacteria;p\_\_Bacteroidetes;c\_\_Bacteroidia;o\_\_Cytophagales | 0 | 0.0% | 0.0% | 0.0% |
|  | k\_\_Bacteria;p\_\_Bacteroidetes;c\_\_Bacteroidia;o\_\_Flavobacteriales | 0 | 0.0% | 0.0% | 0.0% |
|  | k\_\_Bacteria;p\_\_Bacteroidetes;c\_\_Bacteroidia;o\_\_Sphingobacteriales | 0 | 0.0% | 0.0% | 0.0% |
|  | k\_\_Bacteria;p\_\_Deinococcus-Thermus;c\_\_Deinococci;o\_\_Deinococcales | 0 | 0.0% | 0.0% | 0.0% |
|  | k\_\_Bacteria;p\_\_Firmicutes;Other;Other | 0 | 0.0% | 0.0% | 0.0% |
|  | k\_\_Bacteria;p\_\_Firmicutes;c\_\_Bacilli;o\_\_Bacillales | 0 | 0.0% | 0.0% | 0.0% |
|  | k\_\_Bacteria;p\_\_Firmicutes;c\_\_Bacilli;o\_\_Lactobacillales | 0 | 1.0% | 1.1% | 0.9% |
|  | k\_\_Bacteria;p\_\_Firmicutes;c\_\_Clostridia;o\_\_Clostridiales | 1 | 36.6% | 38.2% | 35.0% |
|  | k\_\_Bacteria;p\_\_Firmicutes;c\_\_Erysipelotrichia;o\_\_Erysipelotrichales | 0 | 0.4% | 0.3% | 0.6% |
|  | k\_\_Bacteria;p\_\_Firmicutes;c\_\_Negativicutes;o\_\_Selenomonadales | 0 | 0.0% | 0.0% | 0.1% |
|  | k\_\_Bacteria;p\_\_Fusobacteria;c\_\_Fusobacteriia;o\_\_Fusobacteriales | 0 | 0.0% | 0.0% | 0.0% |
|  | k\_\_Bacteria;p\_\_Melainabacteria;Other;Other | 0 | 0.0% | 0.0% | 0.1% |
|  | k\_\_Bacteria;p\_\_Oxyphotobacteria;c\_\_unidentified\_Oxyphotobacteria;o\_\_unidentified\_Oxyphotobacteria | 0 | 0.0% | 0.1% | 0.0% |
|  | k\_\_Bacteria;p\_\_Proteobacteria;Other;Other | 0 | 0.0% | 0.0% | 0.0% |
|  | k\_\_Bacteria;p\_\_Proteobacteria;c\_\_Alphaproteobacteria;o\_\_Caulobacterales | 0 | 0.0% | 0.0% | 0.0% |
|  | k\_\_Bacteria;p\_\_Proteobacteria;c\_\_Alphaproteobacteria;o\_\_Rhizobiales | 0 | 0.0% | 0.0% | 0.0% |
|  | k\_\_Bacteria;p\_\_Proteobacteria;c\_\_Alphaproteobacteria;o\_\_Rhodospirillales | 0 | 0.0% | 0.0% | 0.0% |
|  | k\_\_Bacteria;p\_\_Proteobacteria;c\_\_Alphaproteobacteria;o\_\_Sphingomonadales | 0 | 0.0% | 0.0% | 0.0% |
|  | k\_\_Bacteria;p\_\_Proteobacteria;c\_\_Alphaproteobacteria;o\_\_unidentified\_Alphaproteobacteria | 0 | 0.0% | 0.0% | 0.0% |
|  | k\_\_Bacteria;p\_\_Proteobacteria;c\_\_Deltaproteobacteria;o\_\_Desulfovibrionales | 0 | 1.1% | 1.1% | 1.1% |
|  | k\_\_Bacteria;p\_\_Proteobacteria;c\_\_Gammaproteobacteria;o\_\_Alteromonadales | 0 | 0.0% | 0.0% | 0.0% |
|  | k\_\_Bacteria;p\_\_Proteobacteria;c\_\_Gammaproteobacteria;o\_\_Enterobacteriales | 0 | 5.7% | 7.1% | 4.3% |
|  | k\_\_Bacteria;p\_\_Proteobacteria;c\_\_Gammaproteobacteria;o\_\_Pasteurellales | 0 | 0.0% | 0.0% | 0.0% |
|  | k\_\_Bacteria;p\_\_Proteobacteria;c\_\_Gammaproteobacteria;o\_\_Pseudomonadales | 0 | 0.0% | 0.0% | 0.0% |
|  | k\_\_Bacteria;p\_\_Proteobacteria;c\_\_Gammaproteobacteria;o\_\_Xanthomonadales | 0 | 0.0% | 0.0% | 0.0% |
|  | k\_\_Bacteria;p\_\_Proteobacteria;c\_\_Gammaproteobacteria;o\_\_unidentified\_Gammaproteobacteria | 0 | 0.1% | 0.1% | 0.0% |
|  | k\_\_Bacteria;p\_\_Tenericutes;c\_\_Mollicutes;o\_\_ | 0 | 0.5% | 0.1% | 0.8% |
|  | k\_\_Bacteria;p\_\_Tenericutes;c\_\_Mollicutes;o\_\_Anaeroplasmatales | 0 | 0.0% | 0.0% | 0.0% |
|  | k\_\_Bacteria;p\_\_Verrucomicrobia;c\_\_Verrucomicrobiae;o\_\_Chthoniobacterales | 0 | 0.0% | 0.0% | 0.0% |
|  | k\_\_Bacteria;p\_\_Verrucomicrobia;c\_\_Verrucomicrobiae;o\_\_Verrucomicrobiales | 0 | 1.4% | 0.4% | 2.3% |
|  | k\_\_Bacteria;p\_\_unidentified\_Bacteria;c\_\_unidentified\_Bacteria;o\_\_Campylobacterales | 0 | 0.0% | 0.0% | 0.0% |
|  | k\_\_Bacteria;p\_\_unidentified\_Bacteria;c\_\_unidentified\_Bacteria;o\_\_unidentified\_Bacteria | 0 | 0.0% | 0.0% | 0.1% |

|  |  |
| --- | --- |
|  | |
| Taxonomy Summary. Current Level: | |
| View Figure (.pdf)  View Legend (.pdf) |  |
|  |  |


View Table (.txt)

|  |  |  |  |  |  |
| --- | --- | --- | --- | --- | --- |
|  | | Total | | MD | MC |
| Legend | Taxonomy | count | % | % | % |
|  | k\_\_Archaea;p\_\_Euryarchaeota;c\_\_Methanobacteria;o\_\_Methanobacteriales;f\_\_Methanobacteriaceae | 0 | 0.0% | 0.0% | 0.0% |
|  | k\_\_Bacteria;Other;Other;Other;Other | 0 | 0.1% | 0.1% | 0.1% |
|  | k\_\_Bacteria;p\_\_Acidobacteria;c\_\_Holophagae;o\_\_;f\_\_ | 0 | 0.0% | 0.0% | 0.0% |
|  | k\_\_Bacteria;p\_\_Acidobacteria;c\_\_unidentified\_Acidobacteria;o\_\_unidentified\_Acidobacteria;f\_\_Pyrinomonadaceae | 0 | 0.0% | 0.0% | 0.0% |
|  | k\_\_Bacteria;p\_\_Acidobacteria;c\_\_unidentified\_Acidobacteria;o\_\_unidentified\_Acidobacteria;f\_\_unidentified\_Acidobacteria | 0 | 0.0% | 0.0% | 0.0% |
|  | k\_\_Bacteria;p\_\_Actinobacteria;c\_\_Acidimicrobiia;Other;Other | 0 | 0.0% | 0.0% | 0.0% |
|  | k\_\_Bacteria;p\_\_Actinobacteria;c\_\_Acidimicrobiia;o\_\_;f\_\_ | 0 | 0.0% | 0.0% | 0.0% |
|  | k\_\_Bacteria;p\_\_Actinobacteria;c\_\_Coriobacteriia;o\_\_Coriobacteriales;f\_\_Atopobiaceae | 0 | 0.0% | 0.0% | 0.0% |
|  | k\_\_Bacteria;p\_\_Actinobacteria;c\_\_Rubrobacteria;o\_\_Rubrobacterales;f\_\_unidentified\_Rubrobacterales | 0 | 0.0% | 0.0% | 0.0% |
|  | k\_\_Bacteria;p\_\_Actinobacteria;c\_\_Thermoleophilia;o\_\_Gaiellales;f\_\_ | 0 | 0.0% | 0.0% | 0.0% |
|  | k\_\_Bacteria;p\_\_Actinobacteria;c\_\_Thermoleophilia;o\_\_Solirubrobacterales;Other | 0 | 0.0% | 0.0% | 0.0% |
|  | k\_\_Bacteria;p\_\_Actinobacteria;c\_\_Thermoleophilia;o\_\_Solirubrobacterales;f\_\_ | 0 | 0.0% | 0.0% | 0.0% |
|  | k\_\_Bacteria;p\_\_Actinobacteria;c\_\_unidentified\_Actinobacteria;o\_\_Actinomycetales;f\_\_Actinomycetaceae | 0 | 0.0% | 0.0% | 0.0% |
|  | k\_\_Bacteria;p\_\_Actinobacteria;c\_\_unidentified\_Actinobacteria;o\_\_Bifidobacteriales;f\_\_Bifidobacteriaceae | 0 | 0.0% | 0.0% | 0.0% |
|  | k\_\_Bacteria;p\_\_Actinobacteria;c\_\_unidentified\_Actinobacteria;o\_\_Corynebacteriales;f\_\_Corynebacteriaceae | 0 | 0.0% | 0.0% | 0.0% |
|  | k\_\_Bacteria;p\_\_Actinobacteria;c\_\_unidentified\_Actinobacteria;o\_\_Corynebacteriales;f\_\_Nocardiaceae | 0 | 0.0% | 0.0% | 0.0% |
|  | k\_\_Bacteria;p\_\_Actinobacteria;c\_\_unidentified\_Actinobacteria;o\_\_Micrococcales;f\_\_Microbacteriaceae | 0 | 0.0% | 0.0% | 0.0% |
|  | k\_\_Bacteria;p\_\_Actinobacteria;c\_\_unidentified\_Actinobacteria;o\_\_Propionibacteriales;f\_\_Nocardioidaceae | 0 | 0.0% | 0.0% | 0.0% |
|  | k\_\_Bacteria;p\_\_Armatimonadetes;c\_\_Fimbriimonadia;o\_\_Fimbriimonadales;f\_\_Fimbriimonadaceae | 0 | 0.0% | 0.0% | 0.0% |
|  | k\_\_Bacteria;p\_\_Bacteroidetes;c\_\_Bacteroidia;o\_\_Bacteroidales;f\_\_Bacteroidaceae | 0 | 17.7% | 16.7% | 18.8% |
|  | k\_\_Bacteria;p\_\_Bacteroidetes;c\_\_Bacteroidia;o\_\_Bacteroidales;f\_\_Barnesiellaceae | 0 | 0.0% | 0.0% | 0.0% |
|  | k\_\_Bacteria;p\_\_Bacteroidetes;c\_\_Bacteroidia;o\_\_Bacteroidales;f\_\_Marinifilaceae | 0 | 0.1% | 0.1% | 0.1% |
|  | k\_\_Bacteria;p\_\_Bacteroidetes;c\_\_Bacteroidia;o\_\_Bacteroidales;f\_\_Muribaculaceae | 1 | 30.5% | 30.8% | 30.3% |
|  | k\_\_Bacteria;p\_\_Bacteroidetes;c\_\_Bacteroidia;o\_\_Bacteroidales;f\_\_Porphyromonadaceae | 0 | 0.0% | 0.0% | 0.0% |
|  | k\_\_Bacteria;p\_\_Bacteroidetes;c\_\_Bacteroidia;o\_\_Bacteroidales;f\_\_Prevotellaceae | 0 | 1.9% | 1.6% | 2.2% |
|  | k\_\_Bacteria;p\_\_Bacteroidetes;c\_\_Bacteroidia;o\_\_Bacteroidales;f\_\_Rikenellaceae | 0 | 1.9% | 1.5% | 2.4% |
|  | k\_\_Bacteria;p\_\_Bacteroidetes;c\_\_Bacteroidia;o\_\_Bacteroidales;f\_\_Tannerellaceae | 0 | 0.6% | 0.5% | 0.7% |
|  | k\_\_Bacteria;p\_\_Bacteroidetes;c\_\_Bacteroidia;o\_\_Chitinophagales;f\_\_Chitinophagaceae | 0 | 0.0% | 0.0% | 0.0% |
|  | k\_\_Bacteria;p\_\_Bacteroidetes;c\_\_Bacteroidia;o\_\_Cytophagales;Other | 0 | 0.0% | 0.0% | 0.0% |
|  | k\_\_Bacteria;p\_\_Bacteroidetes;c\_\_Bacteroidia;o\_\_Flavobacteriales;f\_\_Flavobacteriaceae | 0 | 0.0% | 0.0% | 0.0% |
|  | k\_\_Bacteria;p\_\_Bacteroidetes;c\_\_Bacteroidia;o\_\_Flavobacteriales;f\_\_unidentified\_Flavobacteriales | 0 | 0.0% | 0.0% | 0.0% |
|  | k\_\_Bacteria;p\_\_Bacteroidetes;c\_\_Bacteroidia;o\_\_Sphingobacteriales;f\_\_ | 0 | 0.0% | 0.0% | 0.0% |
|  | k\_\_Bacteria;p\_\_Deinococcus-Thermus;c\_\_Deinococci;o\_\_Deinococcales;f\_\_Deinococcaceae | 0 | 0.0% | 0.0% | 0.0% |
|  | k\_\_Bacteria;p\_\_Firmicutes;Other;Other;Other | 0 | 0.0% | 0.0% | 0.0% |
|  | k\_\_Bacteria;p\_\_Firmicutes;c\_\_Bacilli;o\_\_Bacillales;f\_\_Staphylococcaceae | 0 | 0.0% | 0.0% | 0.0% |
|  | k\_\_Bacteria;p\_\_Firmicutes;c\_\_Bacilli;o\_\_Bacillales;f\_\_unidentified\_Bacillales | 0 | 0.0% | 0.0% | 0.0% |
|  | k\_\_Bacteria;p\_\_Firmicutes;c\_\_Bacilli;o\_\_Lactobacillales;f\_\_Enterococcaceae | 0 | 0.4% | 0.5% | 0.4% |
|  | k\_\_Bacteria;p\_\_Firmicutes;c\_\_Bacilli;o\_\_Lactobacillales;f\_\_Lactobacillaceae | 0 | 0.5% | 0.6% | 0.5% |
|  | k\_\_Bacteria;p\_\_Firmicutes;c\_\_Bacilli;o\_\_Lactobacillales;f\_\_Leuconostocaceae | 0 | 0.0% | 0.0% | 0.0% |
|  | k\_\_Bacteria;p\_\_Firmicutes;c\_\_Bacilli;o\_\_Lactobacillales;f\_\_Streptococcaceae | 0 | 0.0% | 0.0% | 0.0% |
|  | k\_\_Bacteria;p\_\_Firmicutes;c\_\_Clostridia;o\_\_Clostridiales;Other | 0 | 0.4% | 0.3% | 0.4% |
|  | k\_\_Bacteria;p\_\_Firmicutes;c\_\_Clostridia;o\_\_Clostridiales;f\_\_Christensenellaceae | 0 | 0.0% | 0.0% | 0.0% |
|  | k\_\_Bacteria;p\_\_Firmicutes;c\_\_Clostridia;o\_\_Clostridiales;f\_\_Eubacteriaceae | 0 | 0.0% | 0.0% | 0.0% |
|  | k\_\_Bacteria;p\_\_Firmicutes;c\_\_Clostridia;o\_\_Clostridiales;f\_\_Lachnospiraceae | 0 | 23.3% | 29.2% | 17.4% |
|  | k\_\_Bacteria;p\_\_Firmicutes;c\_\_Clostridia;o\_\_Clostridiales;f\_\_Peptococcaceae | 0 | 0.1% | 0.1% | 0.1% |
|  | k\_\_Bacteria;p\_\_Firmicutes;c\_\_Clostridia;o\_\_Clostridiales;f\_\_Peptostreptococcaceae | 0 | 0.0% | 0.0% | 0.0% |
|  | k\_\_Bacteria;p\_\_Firmicutes;c\_\_Clostridia;o\_\_Clostridiales;f\_\_Ruminococcaceae | 0 | 12.7% | 8.5% | 16.9% |
|  | k\_\_Bacteria;p\_\_Firmicutes;c\_\_Clostridia;o\_\_Clostridiales;f\_\_unidentified\_Clostridiales | 0 | 0.1% | 0.1% | 0.2% |
|  | k\_\_Bacteria;p\_\_Firmicutes;c\_\_Erysipelotrichia;o\_\_Erysipelotrichales;f\_\_Erysipelotrichaceae | 0 | 0.4% | 0.3% | 0.6% |
|  | k\_\_Bacteria;p\_\_Firmicutes;c\_\_Negativicutes;o\_\_Selenomonadales;f\_\_Acidaminococcaceae | 0 | 0.0% | 0.0% | 0.0% |
|  | k\_\_Bacteria;p\_\_Firmicutes;c\_\_Negativicutes;o\_\_Selenomonadales;f\_\_Veillonellaceae | 0 | 0.0% | 0.0% | 0.0% |
|  | k\_\_Bacteria;p\_\_Fusobacteria;c\_\_Fusobacteriia;o\_\_Fusobacteriales;f\_\_Fusobacteriaceae | 0 | 0.0% | 0.0% | 0.0% |
|  | k\_\_Bacteria;p\_\_Melainabacteria;Other;Other;Other | 0 | 0.0% | 0.0% | 0.1% |
|  | k\_\_Bacteria;p\_\_Oxyphotobacteria;c\_\_unidentified\_Oxyphotobacteria;o\_\_unidentified\_Oxyphotobacteria;f\_\_unidentified\_Oxyphotobacteria | 0 | 0.0% | 0.1% | 0.0% |
|  | k\_\_Bacteria;p\_\_Proteobacteria;Other;Other;Other | 0 | 0.0% | 0.0% | 0.0% |
|  | k\_\_Bacteria;p\_\_Proteobacteria;c\_\_Alphaproteobacteria;o\_\_Caulobacterales;f\_\_Caulobacteraceae | 0 | 0.0% | 0.0% | 0.0% |
|  | k\_\_Bacteria;p\_\_Proteobacteria;c\_\_Alphaproteobacteria;o\_\_Rhizobiales;Other | 0 | 0.0% | 0.0% | 0.0% |
|  | k\_\_Bacteria;p\_\_Proteobacteria;c\_\_Alphaproteobacteria;o\_\_Rhizobiales;f\_\_Beijerinckiaceae | 0 | 0.0% | 0.0% | 0.0% |
|  | k\_\_Bacteria;p\_\_Proteobacteria;c\_\_Alphaproteobacteria;o\_\_Rhizobiales;f\_\_Rhizobiaceae | 0 | 0.0% | 0.0% | 0.0% |
|  | k\_\_Bacteria;p\_\_Proteobacteria;c\_\_Alphaproteobacteria;o\_\_Rhizobiales;f\_\_Xanthobacteraceae | 0 | 0.0% | 0.0% | 0.0% |
|  | k\_\_Bacteria;p\_\_Proteobacteria;c\_\_Alphaproteobacteria;o\_\_Rhodospirillales;f\_\_unidentified\_Rhodospirillales | 0 | 0.0% | 0.0% | 0.0% |
|  | k\_\_Bacteria;p\_\_Proteobacteria;c\_\_Alphaproteobacteria;o\_\_Sphingomonadales;f\_\_Sphingomonadaceae | 0 | 0.0% | 0.0% | 0.0% |
|  | k\_\_Bacteria;p\_\_Proteobacteria;c\_\_Alphaproteobacteria;o\_\_unidentified\_Alphaproteobacteria;f\_\_unidentified\_Alphaproteobacteria | 0 | 0.0% | 0.0% | 0.0% |
|  | k\_\_Bacteria;p\_\_Proteobacteria;c\_\_Deltaproteobacteria;o\_\_Desulfovibrionales;f\_\_Desulfovibrionaceae | 0 | 1.1% | 1.1% | 1.1% |
|  | k\_\_Bacteria;p\_\_Proteobacteria;c\_\_Gammaproteobacteria;o\_\_Alteromonadales;f\_\_Shewanellaceae | 0 | 0.0% | 0.0% | 0.0% |
|  | k\_\_Bacteria;p\_\_Proteobacteria;c\_\_Gammaproteobacteria;o\_\_Enterobacteriales;f\_\_Enterobacteriaceae | 0 | 5.7% | 7.1% | 4.3% |
|  | k\_\_Bacteria;p\_\_Proteobacteria;c\_\_Gammaproteobacteria;o\_\_Pasteurellales;f\_\_Pasteurellaceae | 0 | 0.0% | 0.0% | 0.0% |
|  | k\_\_Bacteria;p\_\_Proteobacteria;c\_\_Gammaproteobacteria;o\_\_Pseudomonadales;f\_\_Moraxellaceae | 0 | 0.0% | 0.0% | 0.0% |
|  | k\_\_Bacteria;p\_\_Proteobacteria;c\_\_Gammaproteobacteria;o\_\_Pseudomonadales;f\_\_Pseudomonadaceae | 0 | 0.0% | 0.0% | 0.0% |
|  | k\_\_Bacteria;p\_\_Proteobacteria;c\_\_Gammaproteobacteria;o\_\_Xanthomonadales;f\_\_Rhodanobacteraceae | 0 | 0.0% | 0.0% | 0.0% |
|  | k\_\_Bacteria;p\_\_Proteobacteria;c\_\_Gammaproteobacteria;o\_\_Xanthomonadales;f\_\_Xanthomonadaceae | 0 | 0.0% | 0.0% | 0.0% |
|  | k\_\_Bacteria;p\_\_Proteobacteria;c\_\_Gammaproteobacteria;o\_\_unidentified\_Gammaproteobacteria;f\_\_Burkholderiaceae | 0 | 0.0% | 0.1% | 0.0% |
|  | k\_\_Bacteria;p\_\_Proteobacteria;c\_\_Gammaproteobacteria;o\_\_unidentified\_Gammaproteobacteria;f\_\_Neisseriaceae | 0 | 0.0% | 0.0% | 0.0% |
|  | k\_\_Bacteria;p\_\_Tenericutes;c\_\_Mollicutes;o\_\_;f\_\_ | 0 | 0.5% | 0.1% | 0.8% |
|  | k\_\_Bacteria;p\_\_Tenericutes;c\_\_Mollicutes;o\_\_Anaeroplasmatales;f\_\_Anaeroplasmataceae | 0 | 0.0% | 0.0% | 0.0% |
|  | k\_\_Bacteria;p\_\_Verrucomicrobia;c\_\_Verrucomicrobiae;o\_\_Chthoniobacterales;f\_\_Chthoniobacteraceae | 0 | 0.0% | 0.0% | 0.0% |
|  | k\_\_Bacteria;p\_\_Verrucomicrobia;c\_\_Verrucomicrobiae;o\_\_Verrucomicrobiales;f\_\_Akkermansiaceae | 0 | 1.4% | 0.4% | 2.3% |
|  | k\_\_Bacteria;p\_\_unidentified\_Bacteria;c\_\_unidentified\_Bacteria;o\_\_Campylobacterales;f\_\_Campylobacteraceae | 0 | 0.0% | 0.0% | 0.0% |
|  | k\_\_Bacteria;p\_\_unidentified\_Bacteria;c\_\_unidentified\_Bacteria;o\_\_Campylobacterales;f\_\_unidentified\_Campylobacterales | 0 | 0.0% | 0.0% | 0.0% |
|  | k\_\_Bacteria;p\_\_unidentified\_Bacteria;c\_\_unidentified\_Bacteria;o\_\_unidentified\_Bacteria;f\_\_unidentified\_Bacteria | 0 | 0.0% | 0.0% | 0.1% |

|  |  |
| --- | --- |
|  | |
| Taxonomy Summary. Current Level: | |
| View Figure (.pdf)  View Legend (.pdf) |  |
|  |  |


View Table (.txt)

|  |  |  |  |  |  |
| --- | --- | --- | --- | --- | --- |
|  | | Total | | MD | MC |
| Legend | Taxonomy | count | % | % | % |
|  | k\_\_Archaea;p\_\_Euryarchaeota;c\_\_Methanobacteria;o\_\_Methanobacteriales;f\_\_Methanobacteriaceae;g\_\_Methanobrevibacter | 0 | 0.0% | 0.0% | 0.0% |
|  | k\_\_Bacteria;Other;Other;Other;Other;Other | 0 | 0.1% | 0.1% | 0.1% |
|  | k\_\_Bacteria;p\_\_Acidobacteria;c\_\_Holophagae;o\_\_;f\_\_;g\_\_ | 0 | 0.0% | 0.0% | 0.0% |
|  | k\_\_Bacteria;p\_\_Acidobacteria;c\_\_unidentified\_Acidobacteria;o\_\_unidentified\_Acidobacteria;f\_\_Pyrinomonadaceae;g\_\_unidentified\_Pyrinomonadaceae | 0 | 0.0% | 0.0% | 0.0% |
|  | k\_\_Bacteria;p\_\_Acidobacteria;c\_\_unidentified\_Acidobacteria;o\_\_unidentified\_Acidobacteria;f\_\_unidentified\_Acidobacteria;g\_\_Blastocatella | 0 | 0.0% | 0.0% | 0.0% |
|  | k\_\_Bacteria;p\_\_Actinobacteria;c\_\_Acidimicrobiia;Other;Other;Other | 0 | 0.0% | 0.0% | 0.0% |
|  | k\_\_Bacteria;p\_\_Actinobacteria;c\_\_Acidimicrobiia;o\_\_;f\_\_;g\_\_ | 0 | 0.0% | 0.0% | 0.0% |
|  | k\_\_Bacteria;p\_\_Actinobacteria;c\_\_Coriobacteriia;o\_\_Coriobacteriales;f\_\_Atopobiaceae;g\_\_Atopobium | 0 | 0.0% | 0.0% | 0.0% |
|  | k\_\_Bacteria;p\_\_Actinobacteria;c\_\_Rubrobacteria;o\_\_Rubrobacterales;f\_\_unidentified\_Rubrobacterales;g\_\_Rubrobacter | 0 | 0.0% | 0.0% | 0.0% |
|  | k\_\_Bacteria;p\_\_Actinobacteria;c\_\_Thermoleophilia;o\_\_Gaiellales;f\_\_;g\_\_ | 0 | 0.0% | 0.0% | 0.0% |
|  | k\_\_Bacteria;p\_\_Actinobacteria;c\_\_Thermoleophilia;o\_\_Solirubrobacterales;Other;Other | 0 | 0.0% | 0.0% | 0.0% |
|  | k\_\_Bacteria;p\_\_Actinobacteria;c\_\_Thermoleophilia;o\_\_Solirubrobacterales;f\_\_;g\_\_ | 0 | 0.0% | 0.0% | 0.0% |
|  | k\_\_Bacteria;p\_\_Actinobacteria;c\_\_unidentified\_Actinobacteria;o\_\_Actinomycetales;f\_\_Actinomycetaceae;g\_\_Actinomyces | 0 | 0.0% | 0.0% | 0.0% |
|  | k\_\_Bacteria;p\_\_Actinobacteria;c\_\_unidentified\_Actinobacteria;o\_\_Bifidobacteriales;f\_\_Bifidobacteriaceae;g\_\_Bifidobacterium | 0 | 0.0% | 0.0% | 0.0% |
|  | k\_\_Bacteria;p\_\_Actinobacteria;c\_\_unidentified\_Actinobacteria;o\_\_Corynebacteriales;f\_\_Corynebacteriaceae;g\_\_unidentified\_Corynebacteriaceae | 0 | 0.0% | 0.0% | 0.0% |
|  | k\_\_Bacteria;p\_\_Actinobacteria;c\_\_unidentified\_Actinobacteria;o\_\_Corynebacteriales;f\_\_Nocardiaceae;g\_\_Rhodococcus | 0 | 0.0% | 0.0% | 0.0% |
|  | k\_\_Bacteria;p\_\_Actinobacteria;c\_\_unidentified\_Actinobacteria;o\_\_Micrococcales;f\_\_Microbacteriaceae;Other | 0 | 0.0% | 0.0% | 0.0% |
|  | k\_\_Bacteria;p\_\_Actinobacteria;c\_\_unidentified\_Actinobacteria;o\_\_Propionibacteriales;f\_\_Nocardioidaceae;g\_\_Aeromicrobium | 0 | 0.0% | 0.0% | 0.0% |
|  | k\_\_Bacteria;p\_\_Armatimonadetes;c\_\_Fimbriimonadia;o\_\_Fimbriimonadales;f\_\_Fimbriimonadaceae;g\_\_ | 0 | 0.0% | 0.0% | 0.0% |
|  | k\_\_Bacteria;p\_\_Bacteroidetes;c\_\_Bacteroidia;o\_\_Bacteroidales;f\_\_Bacteroidaceae;g\_\_Bacteroides | 0 | 17.7% | 16.7% | 18.8% |
|  | k\_\_Bacteria;p\_\_Bacteroidetes;c\_\_Bacteroidia;o\_\_Bacteroidales;f\_\_Barnesiellaceae;g\_\_Barnesiella | 0 | 0.0% | 0.0% | 0.0% |
|  | k\_\_Bacteria;p\_\_Bacteroidetes;c\_\_Bacteroidia;o\_\_Bacteroidales;f\_\_Barnesiellaceae;g\_\_Coprobacter | 0 | 0.0% | 0.0% | 0.0% |
|  | k\_\_Bacteria;p\_\_Bacteroidetes;c\_\_Bacteroidia;o\_\_Bacteroidales;f\_\_Marinifilaceae;g\_\_Butyricimonas | 0 | 0.1% | 0.1% | 0.1% |
|  | k\_\_Bacteria;p\_\_Bacteroidetes;c\_\_Bacteroidia;o\_\_Bacteroidales;f\_\_Marinifilaceae;g\_\_Odoribacter | 0 | 0.0% | 0.0% | 0.0% |
|  | k\_\_Bacteria;p\_\_Bacteroidetes;c\_\_Bacteroidia;o\_\_Bacteroidales;f\_\_Muribaculaceae;g\_\_ | 1 | 29.8% | 30.1% | 29.5% |
|  | k\_\_Bacteria;p\_\_Bacteroidetes;c\_\_Bacteroidia;o\_\_Bacteroidales;f\_\_Muribaculaceae;g\_\_Muribaculum | 0 | 0.7% | 0.7% | 0.8% |
|  | k\_\_Bacteria;p\_\_Bacteroidetes;c\_\_Bacteroidia;o\_\_Bacteroidales;f\_\_Porphyromonadaceae;g\_\_Porphyromonas | 0 | 0.0% | 0.0% | 0.0% |
|  | k\_\_Bacteria;p\_\_Bacteroidetes;c\_\_Bacteroidia;o\_\_Bacteroidales;f\_\_Prevotellaceae;Other | 0 | 0.3% | 0.0% | 0.6% |
|  | k\_\_Bacteria;p\_\_Bacteroidetes;c\_\_Bacteroidia;o\_\_Bacteroidales;f\_\_Prevotellaceae;g\_\_ | 0 | 0.0% | 0.0% | 0.0% |
|  | k\_\_Bacteria;p\_\_Bacteroidetes;c\_\_Bacteroidia;o\_\_Bacteroidales;f\_\_Prevotellaceae;g\_\_Alloprevotella | 0 | 1.6% | 1.6% | 1.6% |
|  | k\_\_Bacteria;p\_\_Bacteroidetes;c\_\_Bacteroidia;o\_\_Bacteroidales;f\_\_Prevotellaceae;g\_\_Prevotella | 0 | 0.0% | 0.0% | 0.0% |
|  | k\_\_Bacteria;p\_\_Bacteroidetes;c\_\_Bacteroidia;o\_\_Bacteroidales;f\_\_Prevotellaceae;g\_\_unidentified\_Prevotellaceae | 0 | 0.0% | 0.0% | 0.0% |
|  | k\_\_Bacteria;p\_\_Bacteroidetes;c\_\_Bacteroidia;o\_\_Bacteroidales;f\_\_Rikenellaceae;g\_\_Alistipes | 0 | 1.9% | 1.5% | 2.4% |
|  | k\_\_Bacteria;p\_\_Bacteroidetes;c\_\_Bacteroidia;o\_\_Bacteroidales;f\_\_Tannerellaceae;g\_\_Parabacteroides | 0 | 0.6% | 0.5% | 0.7% |
|  | k\_\_Bacteria;p\_\_Bacteroidetes;c\_\_Bacteroidia;o\_\_Chitinophagales;f\_\_Chitinophagaceae;g\_\_Segetibacter | 0 | 0.0% | 0.0% | 0.0% |
|  | k\_\_Bacteria;p\_\_Bacteroidetes;c\_\_Bacteroidia;o\_\_Cytophagales;Other;Other | 0 | 0.0% | 0.0% | 0.0% |
|  | k\_\_Bacteria;p\_\_Bacteroidetes;c\_\_Bacteroidia;o\_\_Flavobacteriales;f\_\_Flavobacteriaceae;Other | 0 | 0.0% | 0.0% | 0.0% |
|  | k\_\_Bacteria;p\_\_Bacteroidetes;c\_\_Bacteroidia;o\_\_Flavobacteriales;f\_\_Flavobacteriaceae;g\_\_Tenacibaculum | 0 | 0.0% | 0.0% | 0.0% |
|  | k\_\_Bacteria;p\_\_Bacteroidetes;c\_\_Bacteroidia;o\_\_Flavobacteriales;f\_\_unidentified\_Flavobacteriales;g\_\_Chryseobacterium | 0 | 0.0% | 0.0% | 0.0% |
|  | k\_\_Bacteria;p\_\_Bacteroidetes;c\_\_Bacteroidia;o\_\_Sphingobacteriales;f\_\_;g\_\_ | 0 | 0.0% | 0.0% | 0.0% |
|  | k\_\_Bacteria;p\_\_Deinococcus-Thermus;c\_\_Deinococci;o\_\_Deinococcales;f\_\_Deinococcaceae;g\_\_Deinococcus | 0 | 0.0% | 0.0% | 0.0% |
|  | k\_\_Bacteria;p\_\_Firmicutes;Other;Other;Other;Other | 0 | 0.0% | 0.0% | 0.0% |
|  | k\_\_Bacteria;p\_\_Firmicutes;c\_\_Bacilli;o\_\_Bacillales;f\_\_Staphylococcaceae;g\_\_Staphylococcus | 0 | 0.0% | 0.0% | 0.0% |
|  | k\_\_Bacteria;p\_\_Firmicutes;c\_\_Bacilli;o\_\_Bacillales;f\_\_unidentified\_Bacillales;g\_\_Exiguobacterium | 0 | 0.0% | 0.0% | 0.0% |
|  | k\_\_Bacteria;p\_\_Firmicutes;c\_\_Bacilli;o\_\_Lactobacillales;f\_\_Enterococcaceae;g\_\_Enterococcus | 0 | 0.4% | 0.5% | 0.4% |
|  | k\_\_Bacteria;p\_\_Firmicutes;c\_\_Bacilli;o\_\_Lactobacillales;f\_\_Lactobacillaceae;g\_\_Lactobacillus | 0 | 0.5% | 0.6% | 0.5% |
|  | k\_\_Bacteria;p\_\_Firmicutes;c\_\_Bacilli;o\_\_Lactobacillales;f\_\_Leuconostocaceae;g\_\_Leuconostoc | 0 | 0.0% | 0.0% | 0.0% |
|  | k\_\_Bacteria;p\_\_Firmicutes;c\_\_Bacilli;o\_\_Lactobacillales;f\_\_Streptococcaceae;g\_\_Streptococcus | 0 | 0.0% | 0.0% | 0.0% |
|  | k\_\_Bacteria;p\_\_Firmicutes;c\_\_Clostridia;o\_\_Clostridiales;Other;Other | 0 | 0.4% | 0.3% | 0.4% |
|  | k\_\_Bacteria;p\_\_Firmicutes;c\_\_Clostridia;o\_\_Clostridiales;f\_\_Christensenellaceae;g\_\_ | 0 | 0.0% | 0.0% | 0.0% |
|  | k\_\_Bacteria;p\_\_Firmicutes;c\_\_Clostridia;o\_\_Clostridiales;f\_\_Eubacteriaceae;g\_\_Anaerofustis | 0 | 0.0% | 0.0% | 0.0% |
|  | k\_\_Bacteria;p\_\_Firmicutes;c\_\_Clostridia;o\_\_Clostridiales;f\_\_Lachnospiraceae;Other | 0 | 14.1% | 17.7% | 10.4% |
|  | k\_\_Bacteria;p\_\_Firmicutes;c\_\_Clostridia;o\_\_Clostridiales;f\_\_Lachnospiraceae;g\_\_ | 0 | 0.5% | 0.6% | 0.4% |
|  | k\_\_Bacteria;p\_\_Firmicutes;c\_\_Clostridia;o\_\_Clostridiales;f\_\_Lachnospiraceae;g\_\_Acetatifactor | 0 | 0.1% | 0.2% | 0.1% |
|  | k\_\_Bacteria;p\_\_Firmicutes;c\_\_Clostridia;o\_\_Clostridiales;f\_\_Lachnospiraceae;g\_\_Agathobacter | 0 | 0.0% | 0.0% | 0.0% |
|  | k\_\_Bacteria;p\_\_Firmicutes;c\_\_Clostridia;o\_\_Clostridiales;f\_\_Lachnospiraceae;g\_\_Anaerostipes | 0 | 0.0% | 0.0% | 0.0% |
|  | k\_\_Bacteria;p\_\_Firmicutes;c\_\_Clostridia;o\_\_Clostridiales;f\_\_Lachnospiraceae;g\_\_Blautia | 0 | 1.1% | 1.6% | 0.7% |
|  | k\_\_Bacteria;p\_\_Firmicutes;c\_\_Clostridia;o\_\_Clostridiales;f\_\_Lachnospiraceae;g\_\_Fusicatenibacter | 0 | 0.0% | 0.0% | 0.0% |
|  | k\_\_Bacteria;p\_\_Firmicutes;c\_\_Clostridia;o\_\_Clostridiales;f\_\_Lachnospiraceae;g\_\_Lachnoclostridium | 0 | 1.3% | 1.4% | 1.1% |
|  | k\_\_Bacteria;p\_\_Firmicutes;c\_\_Clostridia;o\_\_Clostridiales;f\_\_Lachnospiraceae;g\_\_Lachnospira | 0 | 0.0% | 0.0% | 0.0% |
|  | k\_\_Bacteria;p\_\_Firmicutes;c\_\_Clostridia;o\_\_Clostridiales;f\_\_Lachnospiraceae;g\_\_Marvinbryantia | 0 | 0.0% | 0.0% | 0.0% |
|  | k\_\_Bacteria;p\_\_Firmicutes;c\_\_Clostridia;o\_\_Clostridiales;f\_\_Lachnospiraceae;g\_\_Roseburia | 0 | 1.2% | 1.5% | 0.8% |
|  | k\_\_Bacteria;p\_\_Firmicutes;c\_\_Clostridia;o\_\_Clostridiales;f\_\_Lachnospiraceae;g\_\_Tyzzerella | 0 | 0.0% | 0.1% | 0.0% |
|  | k\_\_Bacteria;p\_\_Firmicutes;c\_\_Clostridia;o\_\_Clostridiales;f\_\_Lachnospiraceae;g\_\_unidentified\_Lachnospiraceae | 0 | 5.0% | 6.3% | 3.8% |
|  | k\_\_Bacteria;p\_\_Firmicutes;c\_\_Clostridia;o\_\_Clostridiales;f\_\_Peptococcaceae;Other | 0 | 0.0% | 0.0% | 0.0% |
|  | k\_\_Bacteria;p\_\_Firmicutes;c\_\_Clostridia;o\_\_Clostridiales;f\_\_Peptococcaceae;g\_\_ | 0 | 0.1% | 0.1% | 0.1% |
|  | k\_\_Bacteria;p\_\_Firmicutes;c\_\_Clostridia;o\_\_Clostridiales;f\_\_Peptococcaceae;g\_\_Peptococcus | 0 | 0.0% | 0.0% | 0.0% |
|  | k\_\_Bacteria;p\_\_Firmicutes;c\_\_Clostridia;o\_\_Clostridiales;f\_\_Peptostreptococcaceae;g\_\_Clostridioides | 0 | 0.0% | 0.0% | 0.0% |
|  | k\_\_Bacteria;p\_\_Firmicutes;c\_\_Clostridia;o\_\_Clostridiales;f\_\_Peptostreptococcaceae;g\_\_Peptostreptococcus | 0 | 0.0% | 0.0% | 0.0% |
|  | k\_\_Bacteria;p\_\_Firmicutes;c\_\_Clostridia;o\_\_Clostridiales;f\_\_Peptostreptococcaceae;g\_\_Romboutsia | 0 | 0.0% | 0.0% | 0.0% |
|  | k\_\_Bacteria;p\_\_Firmicutes;c\_\_Clostridia;o\_\_Clostridiales;f\_\_Ruminococcaceae;Other | 0 | 6.3% | 3.4% | 9.3% |
|  | k\_\_Bacteria;p\_\_Firmicutes;c\_\_Clostridia;o\_\_Clostridiales;f\_\_Ruminococcaceae;g\_\_Anaerotruncus | 0 | 0.3% | 0.3% | 0.2% |
|  | k\_\_Bacteria;p\_\_Firmicutes;c\_\_Clostridia;o\_\_Clostridiales;f\_\_Ruminococcaceae;g\_\_Butyricicoccus | 0 | 0.4% | 0.5% | 0.4% |
|  | k\_\_Bacteria;p\_\_Firmicutes;c\_\_Clostridia;o\_\_Clostridiales;f\_\_Ruminococcaceae;g\_\_Caproiciproducens | 0 | 0.0% | 0.0% | 0.0% |
|  | k\_\_Bacteria;p\_\_Firmicutes;c\_\_Clostridia;o\_\_Clostridiales;f\_\_Ruminococcaceae;g\_\_Faecalibacterium | 0 | 0.1% | 0.1% | 0.1% |
|  | k\_\_Bacteria;p\_\_Firmicutes;c\_\_Clostridia;o\_\_Clostridiales;f\_\_Ruminococcaceae;g\_\_Flavonifractor | 0 | 0.0% | 0.0% | 0.0% |
|  | k\_\_Bacteria;p\_\_Firmicutes;c\_\_Clostridia;o\_\_Clostridiales;f\_\_Ruminococcaceae;g\_\_Harryflintia | 0 | 0.1% | 0.1% | 0.1% |
|  | k\_\_Bacteria;p\_\_Firmicutes;c\_\_Clostridia;o\_\_Clostridiales;f\_\_Ruminococcaceae;g\_\_Hydrogenoanaerobacterium | 0 | 0.0% | 0.0% | 0.0% |
|  | k\_\_Bacteria;p\_\_Firmicutes;c\_\_Clostridia;o\_\_Clostridiales;f\_\_Ruminococcaceae;g\_\_Intestinimonas | 0 | 0.3% | 0.2% | 0.3% |
|  | k\_\_Bacteria;p\_\_Firmicutes;c\_\_Clostridia;o\_\_Clostridiales;f\_\_Ruminococcaceae;g\_\_Oscillibacter | 0 | 0.7% | 0.4% | 0.9% |
|  | k\_\_Bacteria;p\_\_Firmicutes;c\_\_Clostridia;o\_\_Clostridiales;f\_\_Ruminococcaceae;g\_\_Ruminiclostridium | 0 | 0.5% | 0.6% | 0.4% |
|  | k\_\_Bacteria;p\_\_Firmicutes;c\_\_Clostridia;o\_\_Clostridiales;f\_\_Ruminococcaceae;g\_\_Subdoligranulum | 0 | 0.0% | 0.0% | 0.0% |
|  | k\_\_Bacteria;p\_\_Firmicutes;c\_\_Clostridia;o\_\_Clostridiales;f\_\_Ruminococcaceae;g\_\_unidentified\_Ruminococcaceae | 0 | 4.0% | 2.9% | 5.2% |
|  | k\_\_Bacteria;p\_\_Firmicutes;c\_\_Clostridia;o\_\_Clostridiales;f\_\_unidentified\_Clostridiales;Other | 0 | 0.0% | 0.0% | 0.0% |
|  | k\_\_Bacteria;p\_\_Firmicutes;c\_\_Clostridia;o\_\_Clostridiales;f\_\_unidentified\_Clostridiales;g\_\_Anaerovorax | 0 | 0.0% | 0.0% | 0.0% |
|  | k\_\_Bacteria;p\_\_Firmicutes;c\_\_Clostridia;o\_\_Clostridiales;f\_\_unidentified\_Clostridiales;g\_\_Parvimonas | 0 | 0.0% | 0.0% | 0.0% |
|  | k\_\_Bacteria;p\_\_Firmicutes;c\_\_Clostridia;o\_\_Clostridiales;f\_\_unidentified\_Clostridiales;g\_\_unidentified\_Clostridiales | 0 | 0.1% | 0.1% | 0.1% |
|  | k\_\_Bacteria;p\_\_Firmicutes;c\_\_Erysipelotrichia;o\_\_Erysipelotrichales;f\_\_Erysipelotrichaceae;Other | 0 | 0.2% | 0.1% | 0.3% |
|  | k\_\_Bacteria;p\_\_Firmicutes;c\_\_Erysipelotrichia;o\_\_Erysipelotrichales;f\_\_Erysipelotrichaceae;g\_\_Dubosiella | 0 | 0.2% | 0.1% | 0.2% |
|  | k\_\_Bacteria;p\_\_Firmicutes;c\_\_Erysipelotrichia;o\_\_Erysipelotrichales;f\_\_Erysipelotrichaceae;g\_\_Erysipelatoclostridium | 0 | 0.0% | 0.0% | 0.0% |
|  | k\_\_Bacteria;p\_\_Firmicutes;c\_\_Erysipelotrichia;o\_\_Erysipelotrichales;f\_\_Erysipelotrichaceae;g\_\_Faecalitalea | 0 | 0.0% | 0.0% | 0.0% |
|  | k\_\_Bacteria;p\_\_Firmicutes;c\_\_Erysipelotrichia;o\_\_Erysipelotrichales;f\_\_Erysipelotrichaceae;g\_\_Holdemania | 0 | 0.0% | 0.0% | 0.0% |
|  | k\_\_Bacteria;p\_\_Firmicutes;c\_\_Erysipelotrichia;o\_\_Erysipelotrichales;f\_\_Erysipelotrichaceae;g\_\_Turicibacter | 0 | 0.0% | 0.0% | 0.0% |
|  | k\_\_Bacteria;p\_\_Firmicutes;c\_\_Erysipelotrichia;o\_\_Erysipelotrichales;f\_\_Erysipelotrichaceae;g\_\_unidentified\_Erysipelotrichaceae | 0 | 0.0% | 0.0% | 0.0% |
|  | k\_\_Bacteria;p\_\_Firmicutes;c\_\_Negativicutes;o\_\_Selenomonadales;f\_\_Acidaminococcaceae;g\_\_Acidaminococcus | 0 | 0.0% | 0.0% | 0.0% |
|  | k\_\_Bacteria;p\_\_Firmicutes;c\_\_Negativicutes;o\_\_Selenomonadales;f\_\_Acidaminococcaceae;g\_\_Phascolarctobacterium | 0 | 0.0% | 0.0% | 0.0% |
|  | k\_\_Bacteria;p\_\_Firmicutes;c\_\_Negativicutes;o\_\_Selenomonadales;f\_\_Veillonellaceae;g\_\_Allisonella | 0 | 0.0% | 0.0% | 0.0% |
|  | k\_\_Bacteria;p\_\_Firmicutes;c\_\_Negativicutes;o\_\_Selenomonadales;f\_\_Veillonellaceae;g\_\_Dialister | 0 | 0.0% | 0.0% | 0.0% |
|  | k\_\_Bacteria;p\_\_Firmicutes;c\_\_Negativicutes;o\_\_Selenomonadales;f\_\_Veillonellaceae;g\_\_Megamonas | 0 | 0.0% | 0.0% | 0.0% |
|  | k\_\_Bacteria;p\_\_Firmicutes;c\_\_Negativicutes;o\_\_Selenomonadales;f\_\_Veillonellaceae;g\_\_Megasphaera | 0 | 0.0% | 0.0% | 0.0% |
|  | k\_\_Bacteria;p\_\_Firmicutes;c\_\_Negativicutes;o\_\_Selenomonadales;f\_\_Veillonellaceae;g\_\_Veillonella | 0 | 0.0% | 0.0% | 0.0% |
|  | k\_\_Bacteria;p\_\_Fusobacteria;c\_\_Fusobacteriia;o\_\_Fusobacteriales;f\_\_Fusobacteriaceae;Other | 0 | 0.0% | 0.0% | 0.0% |
|  | k\_\_Bacteria;p\_\_Fusobacteria;c\_\_Fusobacteriia;o\_\_Fusobacteriales;f\_\_Fusobacteriaceae;g\_\_Fusobacterium | 0 | 0.0% | 0.0% | 0.0% |
|  | k\_\_Bacteria;p\_\_Melainabacteria;Other;Other;Other;Other | 0 | 0.0% | 0.0% | 0.1% |
|  | k\_\_Bacteria;p\_\_Oxyphotobacteria;c\_\_unidentified\_Oxyphotobacteria;o\_\_unidentified\_Oxyphotobacteria;f\_\_unidentified\_Oxyphotobacteria;g\_\_unidentified\_Oxyphotobacteria | 0 | 0.0% | 0.1% | 0.0% |
|  | k\_\_Bacteria;p\_\_Proteobacteria;Other;Other;Other;Other | 0 | 0.0% | 0.0% | 0.0% |
|  | k\_\_Bacteria;p\_\_Proteobacteria;c\_\_Alphaproteobacteria;o\_\_Caulobacterales;f\_\_Caulobacteraceae;g\_\_Brevundimonas | 0 | 0.0% | 0.0% | 0.0% |
|  | k\_\_Bacteria;p\_\_Proteobacteria;c\_\_Alphaproteobacteria;o\_\_Rhizobiales;Other;Other | 0 | 0.0% | 0.0% | 0.0% |
|  | k\_\_Bacteria;p\_\_Proteobacteria;c\_\_Alphaproteobacteria;o\_\_Rhizobiales;f\_\_Beijerinckiaceae;g\_\_Methylobacterium | 0 | 0.0% | 0.0% | 0.0% |
|  | k\_\_Bacteria;p\_\_Proteobacteria;c\_\_Alphaproteobacteria;o\_\_Rhizobiales;f\_\_Rhizobiaceae;g\_\_Brucella | 0 | 0.0% | 0.0% | 0.0% |
|  | k\_\_Bacteria;p\_\_Proteobacteria;c\_\_Alphaproteobacteria;o\_\_Rhizobiales;f\_\_Rhizobiaceae;g\_\_Ensifer | 0 | 0.0% | 0.0% | 0.0% |
|  | k\_\_Bacteria;p\_\_Proteobacteria;c\_\_Alphaproteobacteria;o\_\_Rhizobiales;f\_\_Rhizobiaceae;g\_\_Phyllobacterium | 0 | 0.0% | 0.0% | 0.0% |
|  | k\_\_Bacteria;p\_\_Proteobacteria;c\_\_Alphaproteobacteria;o\_\_Rhizobiales;f\_\_Xanthobacteraceae;g\_\_Bradyrhizobium | 0 | 0.0% | 0.0% | 0.0% |
|  | k\_\_Bacteria;p\_\_Proteobacteria;c\_\_Alphaproteobacteria;o\_\_Rhodospirillales;f\_\_unidentified\_Rhodospirillales;g\_\_Defluviicoccus | 0 | 0.0% | 0.0% | 0.0% |
|  | k\_\_Bacteria;p\_\_Proteobacteria;c\_\_Alphaproteobacteria;o\_\_Rhodospirillales;f\_\_unidentified\_Rhodospirillales;g\_\_Telmatospirillum | 0 | 0.0% | 0.0% | 0.0% |
|  | k\_\_Bacteria;p\_\_Proteobacteria;c\_\_Alphaproteobacteria;o\_\_Sphingomonadales;f\_\_Sphingomonadaceae;g\_\_Novosphingobium | 0 | 0.0% | 0.0% | 0.0% |
|  | k\_\_Bacteria;p\_\_Proteobacteria;c\_\_Alphaproteobacteria;o\_\_Sphingomonadales;f\_\_Sphingomonadaceae;g\_\_Sphingomonas | 0 | 0.0% | 0.0% | 0.0% |
|  | k\_\_Bacteria;p\_\_Proteobacteria;c\_\_Alphaproteobacteria;o\_\_unidentified\_Alphaproteobacteria;f\_\_unidentified\_Alphaproteobacteria;g\_\_Dongia | 0 | 0.0% | 0.0% | 0.0% |
|  | k\_\_Bacteria;p\_\_Proteobacteria;c\_\_Alphaproteobacteria;o\_\_unidentified\_Alphaproteobacteria;f\_\_unidentified\_Alphaproteobacteria;g\_\_Reyranella | 0 | 0.0% | 0.0% | 0.0% |
|  | k\_\_Bacteria;p\_\_Proteobacteria;c\_\_Deltaproteobacteria;o\_\_Desulfovibrionales;f\_\_Desulfovibrionaceae;g\_\_ | 0 | 0.0% | 0.0% | 0.0% |
|  | k\_\_Bacteria;p\_\_Proteobacteria;c\_\_Deltaproteobacteria;o\_\_Desulfovibrionales;f\_\_Desulfovibrionaceae;g\_\_Bilophila | 0 | 0.0% | 0.0% | 0.0% |
|  | k\_\_Bacteria;p\_\_Proteobacteria;c\_\_Deltaproteobacteria;o\_\_Desulfovibrionales;f\_\_Desulfovibrionaceae;g\_\_Desulfovibrio | 0 | 1.1% | 1.1% | 1.1% |
|  | k\_\_Bacteria;p\_\_Proteobacteria;c\_\_Gammaproteobacteria;o\_\_Alteromonadales;f\_\_Shewanellaceae;g\_\_Shewanella | 0 | 0.0% | 0.0% | 0.0% |
|  | k\_\_Bacteria;p\_\_Proteobacteria;c\_\_Gammaproteobacteria;o\_\_Enterobacteriales;f\_\_Enterobacteriaceae;Other | 0 | 1.3% | 1.5% | 1.2% |
|  | k\_\_Bacteria;p\_\_Proteobacteria;c\_\_Gammaproteobacteria;o\_\_Enterobacteriales;f\_\_Enterobacteriaceae;g\_\_Morganella | 0 | 0.0% | 0.0% | 0.0% |
|  | k\_\_Bacteria;p\_\_Proteobacteria;c\_\_Gammaproteobacteria;o\_\_Enterobacteriales;f\_\_Enterobacteriaceae;g\_\_Proteus | 0 | 0.6% | 0.7% | 0.5% |
|  | k\_\_Bacteria;p\_\_Proteobacteria;c\_\_Gammaproteobacteria;o\_\_Enterobacteriales;f\_\_Enterobacteriaceae;g\_\_unidentified\_Enterobacteriaceae | 0 | 3.7% | 4.9% | 2.6% |
|  | k\_\_Bacteria;p\_\_Proteobacteria;c\_\_Gammaproteobacteria;o\_\_Pasteurellales;f\_\_Pasteurellaceae;Other | 0 | 0.0% | 0.0% | 0.0% |
|  | k\_\_Bacteria;p\_\_Proteobacteria;c\_\_Gammaproteobacteria;o\_\_Pseudomonadales;f\_\_Moraxellaceae;g\_\_Acinetobacter | 0 | 0.0% | 0.0% | 0.0% |
|  | k\_\_Bacteria;p\_\_Proteobacteria;c\_\_Gammaproteobacteria;o\_\_Pseudomonadales;f\_\_Pseudomonadaceae;g\_\_Pseudomonas | 0 | 0.0% | 0.0% | 0.0% |
|  | k\_\_Bacteria;p\_\_Proteobacteria;c\_\_Gammaproteobacteria;o\_\_Xanthomonadales;f\_\_Rhodanobacteraceae;g\_\_Chujaibacter | 0 | 0.0% | 0.0% | 0.0% |
|  | k\_\_Bacteria;p\_\_Proteobacteria;c\_\_Gammaproteobacteria;o\_\_Xanthomonadales;f\_\_Rhodanobacteraceae;g\_\_Rhodanobacter | 0 | 0.0% | 0.0% | 0.0% |
|  | k\_\_Bacteria;p\_\_Proteobacteria;c\_\_Gammaproteobacteria;o\_\_Xanthomonadales;f\_\_Xanthomonadaceae;g\_\_Arenimonas | 0 | 0.0% | 0.0% | 0.0% |
|  | k\_\_Bacteria;p\_\_Proteobacteria;c\_\_Gammaproteobacteria;o\_\_Xanthomonadales;f\_\_Xanthomonadaceae;g\_\_Stenotrophomonas | 0 | 0.0% | 0.0% | 0.0% |
|  | k\_\_Bacteria;p\_\_Proteobacteria;c\_\_Gammaproteobacteria;o\_\_unidentified\_Gammaproteobacteria;f\_\_Burkholderiaceae;Other | 0 | 0.0% | 0.0% | 0.0% |
|  | k\_\_Bacteria;p\_\_Proteobacteria;c\_\_Gammaproteobacteria;o\_\_unidentified\_Gammaproteobacteria;f\_\_Burkholderiaceae;g\_\_Achromobacter | 0 | 0.0% | 0.0% | 0.0% |
|  | k\_\_Bacteria;p\_\_Proteobacteria;c\_\_Gammaproteobacteria;o\_\_unidentified\_Gammaproteobacteria;f\_\_Burkholderiaceae;g\_\_Delftia | 0 | 0.0% | 0.0% | 0.0% |
|  | k\_\_Bacteria;p\_\_Proteobacteria;c\_\_Gammaproteobacteria;o\_\_unidentified\_Gammaproteobacteria;f\_\_Burkholderiaceae;g\_\_Diaphorobacter | 0 | 0.0% | 0.0% | 0.0% |
|  | k\_\_Bacteria;p\_\_Proteobacteria;c\_\_Gammaproteobacteria;o\_\_unidentified\_Gammaproteobacteria;f\_\_Burkholderiaceae;g\_\_Parasutterella | 0 | 0.0% | 0.0% | 0.0% |
|  | k\_\_Bacteria;p\_\_Proteobacteria;c\_\_Gammaproteobacteria;o\_\_unidentified\_Gammaproteobacteria;f\_\_Burkholderiaceae;g\_\_Ralstonia | 0 | 0.0% | 0.0% | 0.0% |
|  | k\_\_Bacteria;p\_\_Proteobacteria;c\_\_Gammaproteobacteria;o\_\_unidentified\_Gammaproteobacteria;f\_\_Burkholderiaceae;g\_\_Sutterella | 0 | 0.0% | 0.0% | 0.0% |
|  | k\_\_Bacteria;p\_\_Proteobacteria;c\_\_Gammaproteobacteria;o\_\_unidentified\_Gammaproteobacteria;f\_\_Neisseriaceae;g\_\_Neisseria | 0 | 0.0% | 0.0% | 0.0% |
|  | k\_\_Bacteria;p\_\_Tenericutes;c\_\_Mollicutes;o\_\_;f\_\_;g\_\_ | 0 | 0.5% | 0.1% | 0.8% |
|  | k\_\_Bacteria;p\_\_Tenericutes;c\_\_Mollicutes;o\_\_Anaeroplasmatales;f\_\_Anaeroplasmataceae;g\_\_Anaeroplasma | 0 | 0.0% | 0.0% | 0.0% |
|  | k\_\_Bacteria;p\_\_Verrucomicrobia;c\_\_Verrucomicrobiae;o\_\_Chthoniobacterales;f\_\_Chthoniobacteraceae;Other | 0 | 0.0% | 0.0% | 0.0% |
|  | k\_\_Bacteria;p\_\_Verrucomicrobia;c\_\_Verrucomicrobiae;o\_\_Verrucomicrobiales;f\_\_Akkermansiaceae;g\_\_Akkermansia | 0 | 1.4% | 0.4% | 2.3% |
|  | k\_\_Bacteria;p\_\_unidentified\_Bacteria;c\_\_unidentified\_Bacteria;o\_\_Campylobacterales;f\_\_Campylobacteraceae;g\_\_Campylobacter | 0 | 0.0% | 0.0% | 0.0% |
|  | k\_\_Bacteria;p\_\_unidentified\_Bacteria;c\_\_unidentified\_Bacteria;o\_\_Campylobacterales;f\_\_unidentified\_Campylobacterales;g\_\_Sulfurospirillum | 0 | 0.0% | 0.0% | 0.0% |
|  | k\_\_Bacteria;p\_\_unidentified\_Bacteria;c\_\_unidentified\_Bacteria;o\_\_unidentified\_Bacteria;f\_\_unidentified\_Bacteria;g\_\_Candidatus\_Saccharimonas | 0 | 0.0% | 0.0% | 0.1% |
|  | k\_\_Bacteria;p\_\_unidentified\_Bacteria;c\_\_unidentified\_Bacteria;o\_\_unidentified\_Bacteria;f\_\_unidentified\_Bacteria;g\_\_unidentified\_Bacteria | 0 | 0.0% | 0.0% | 0.0% |
